# Mean platelet volume and response to the first line therapy in newly diagnosed adult immune thrombocytopenia patients: a retrospective study

**DOI:** 10.3906/sag-1912-242

**Published:** 2020-06-23

**Authors:** Erman AKKUŞ, Çiğdem FİDAN, Gülşah DEMİRCİ, Ali Aytuğ KUŞTAŞ, Meltem KURT YÜKSEL

**Affiliations:** 1 Department of Haematology, Faculty of Medicine, Ankara University, Ankara Turkey; 2 Department of Medical Biochemistry, Faculty of Medicine, Ankara University, Ankara Turkey

**Keywords:** Immune thrombocytopenia, first line treatment, response, mean platelet volume

## Abstract

**Background/aim:**

Immune thrombocytopenia (ITP) is treated by corticosteroids and/or intravenous immune globulin as the first line treatment when necessary. Mean platelet volume (MPV) is a marker of platelet production and function. In this study, we aimed to search the relationship between the MPV and the treatment response in ITP patients and it was hypothesized that MPV can be used as a predictor of the response.

**Materials and methods:**

The 70 newly diagnosed adult primary ITP patients and 70 of healthy people were included. MPV between ITP and healthy population, MPV in the diagnosis and after the treatment between the responders and the nonresponders were compared.

**Results:**

The responders had significantly higher MPV and the nonresponders had significantly lower MPV than the healthy population (11.09 and 10.21 fL, P = 0.03; 9.38 and 10.21 fL, P = 0.001). MPV in the diagnosis was significantly higher in the responders than the nonresponders (11.09 and 9.38 fL, P = 0.005). MPV significantly changed after the treatment in the responders (11.09 to 9.32 fL, P = 0.004).

**Conclusion:**

MPV can be used as a predictor of early response to the first line treatment in newly diagnosed adult primary ITP patients.

## 1. Introduction

Immune thrombocytopenia (ITP) is an acquired thrombocytopenia which is defined by the platelet count (PLT) is less than 100 × 109/L. Primary ITP is caused by antibody and T cell mediated destruction of the platelets and impaired megakaryopoiesis, whereas the secondary ITP is due to underlying disorders. Due to the immune pathophysiological mechanism of the primary ITP, patients are initially treated by corticosteroids and intravenous immune globulin (IVIG), when they need the treatment [1]. Although the platelet count increases in 3–5 days after the treatment, there is no available study that may investigate the predictive factors of the short-term response to the first line therapy. Mean platelet volume (MPV), which is easily available in clinical practice, is a marker of platelet function [2]. Several studies have investigated the MPV value in the diagnosis and differential diagnosis of the ITP and hypoproliferative thrombocytopenia [3–8], relapse of the ITP [9,10] and thrombocytosis [11]. Although these studies have revealed important aspects of the MPV and ITP, they have not investigated the relationship between the MPV and short-term treatment response to the first line therapy. Moreover, some have insufficient study design.

In this study, a retrospective search was implemented in newly diagnosed adult primary ITP patients. We aimed to search the relationship between the MPV and response to the first line therapy hypothesizing the MPV as a possible predictive factor of the treatment response.

## 2. Materials and methods

### 2.1. Subjects

A power calculation (GPower 3.1 software) indicated the need for 30 patients to have 80% power to detect a 2 fL difference in MPV (for example, 10 fL in one group and 12 in the other, with the standard deviation of 3). Difference and standard deviation were chosen according to ITP and healthy population values in the previous studies [6,12].

Patients were retrospectively screened in the patient database of Ankara University, Faculty of Medicine between the January of 2014 and the June of 2017. The last recommendations of the American Society of Haematology and International Working Group were applied for the diagnosis, treatment, and response evaluation [1].

Briefly, the diagnosis of ITP was made by evaluation of history, examination of the patient, review of the peripheral smear and with some additional limited tests (HBsAg, Anti-HCV, Anti-HIV and H. pylori antigen stool tests, direct antiglobulin testing, thyroid function tests). Bone marrow examination was performed when it was needed.

**Figure 1:**
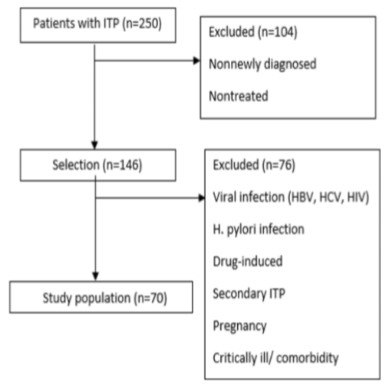
Selection of the study population.

A total of 250 patients diagnosed with ITP were screened in the database. The newly diagnosed outpatient ITP patients (diagnosed less than 3 months), treated by the first line treatment (methylprednisolone, intravenous immune globulin) and patients whose planned data were available were included. Secondary ITP, drug induced thrombocytopenia, HBsAg, anti-HCV, anti-HIV positive and H. pylori antigen positive patients, pregnant and hospitalized patients, patients with severe organ dysfunction, patients using any medication regularly and patients having comorbid disease that can affect the MPV (diabetes mellitus, thyroid dysfunction, renal, liver, and cardiovascular disease) were excluded (Figure 1).The patients who were given platelet transfusion at the presentation were not excluded (because platelet life span is 7–10 days and transfused platelets have shorter life span. In this study, MPV measurements were made before transfusion and at least 1 week after the transfusion.)

Patients were treated by oral corticosteroid (1 mg/kg/d methylprednisolone) or corticosteroid plus 1 g/kg/d IVIG for 2 days. IVIG was initiated on the day of the treatment decision together with corticosteroid and corticosteroid was continued after the IVIG until the second visit for the response evaluation. The treatment decision was made by the responsible doctor at that time. The indications for the treatment were low platelet count and/or clinically significant bleeding.

The response was evaluated beginning from 1 until the 3 weeks after treatment initiation in the outpatient visit. Patients were grouped as responders and nonresponders according to the 1 of 2 response criteria: 1) platelet counts > 100 × 109/L measured on 2 occasions 7 days apart, 2) platelet counts > 30 × 109/L and a greater than twofold increase in platelet count from baseline, measured on 2 occasions 7 days apart.

An independent population of healthy people was included as a control group.

Approval of ethical committee was obtained from Ethics Committee of Ankara University Faculty of Medicine in compliance with the Helsinki Declaration.

### 2.2. Methods

MPV was measured in the diagnosis and response evaluation. Tourniquets were used for drawing blood and collections were completed in less than 1 min. Venous blood was collected into 2 mL EDTA tube and analysed in less than 2 h. Platelet count and MPV were measured by the implementation of 2-angle laser light scattering method in ADVIA 2120i Hematology System (Siemens Healthcare Diagnostics, Tarrytown, NY, USA). Coefficient of variation (both within and between assays) was 1.4%. Internal quality control procedure (ADVIA TESTpoint Hematology Controls) includes 3 different levels (low, normal, and high) and was assessed according to Westgard Rules. The reference ranges in the lab for platelet count and MPV were 150–400 × 109/L and 6.5–10.5 fL, respectively. 

The age, sex, presenting symptom, baseline PLT and MPV in the diagnosis and PLT, MPV in the response evaluation were recorded.

The baseline PLT, MPV between the ITP and healthy population; PLT, MPV between the responders and the nonresponders in baseline and after the treatment; the MPV changes after the treatment in the responder and nonresponders, sensitivity, specificity, positive and negative predictive value of the MPV were compared and evaluated.

### 2.3. Statistical analyses

The median (minimum and maximum), mean (standard deviation (SD)), number and percentage were used as descriptive statistics. The difference in means between 2 groups was evaluated by Student’s t test. The chi square test was used for the difference between proportions. Paired-samples T test was used to evaluate the difference between before and after the treatment measurements. Receiver operating characteristic (ROC) curve was used to describe the performance of a diagnostic test. The area under the corresponding curve was calculated by Hanley and McNeil [13]. The sensitivity and specificity were calculated under various cut-off value. All P values were based on a 2-tailed test of significance (P = 0.05). Analyses were made by using SPSS version 22 (IBM Corp., Armonk, NY, USA).

## 3. Results

### 3.1. Patients and characteristics

Among the 250 patients screened, 70 patients were included in the study according to the including-excluding criteria (Figure 1).

The age, sex, presenting symptom, PLT and MPV in the diagnosis of the study and healthy population are given in Table 1. The median age of the ITP group was 41 (19–80) and there was female dominancy (62.9%). Fifty-three percent of the patients were presented by skin bleeding (petechiae, purpura, and ecchymosis), the 31% was detected incidentally. Mucosal bleeding (epistaxis, gum bleeding, gastrointestinal bleeding) was 13%. The 51% of the patients were also given the IVIG therapy at the beginning of corticosteroid treatment (Table 1).

**Table 1:** Baseline characteristics of the ITP patients and the healthy population.

	Study population(n = 70)	Healthy population (n = 70)	P
Age, year (median, min–max)	41(19–80)	40.5 (21–89)	0.74
Female, n (%)	44 (62.9%)	34 (48.6%)	0.08
Male, n (%)	26 (37.1%)	36 (51.4%)	
PLT, × 109/L (median, min–max)	18 (1 - 63)	260 (157 - 373)	< 0.001
MPV, fL (mean, SD)	10.11 (2.56)	10.21 (0.91)	0.76
Skin bleeding, n (%)	37 (53%)		
Mucosal bleeding, n (%)	9 (13%)		
Organ bleeding, n (%)	2 (3%)		
Incidental, n (%)	22 (31%)		
Plus IVIG, n (%)	36 (51%)		
Total response rate (%) Responder, n Nonresponder, n	43% 30 40		

PLT: platelet, L: litre, MPV: mean platelet volume, fL: femtoliter, IVIG: Intravenous immunoglobulin.

### 3.2. ITP/healthy population

We first evaluated the MPV in the ITP and healthy groups. The median platelet count was 18 × 109/L (1–63 × 109 / L) and MPV was 10 fL (4.8–21.6) in the ITP group. Median MPV was 10.2 fL (7.8–12.3) in the healthy group. The age, sex, and MPV were not significantly different between the ITP and healthy group (P = 0.76 for the MPV, CI: –0.74–0.54) (Table 1). We evaluated the MPV in the diagnosis of responders and nonresponders separately comparing with the healthy population. The responders had significantly higher MPV than the healthy population (11.09 and 10.21, P = 0.03, CI: 0.71–1.68). The nonresponders had significantly lower MPV than the healthy population (8.95 and 10.21, P = 0.001, CI: 0.32–1.33).

### 3.3. Responders/nonresponders

Secondly, we evaluated the MPV difference between the responders and the nonresponders in the diagnosis and after the treatment, respectively. The 30 of 70 patients (43%) responded the treatment according to response criteria. There was not any difference in age, sex, and platelet count in the diagnosis and no difference in the IVIG treatment between the responders and the nonresponders. However, MPV in the diagnosis was significantly higher in the responders than the nonresponders (11.09 and 9.38 fL, P = 0.005, CI: 0.53–2.88). There was not any difference in MPV after the treatment between the responders and the nonresponders (9.32 and 9.4 fL, P = 0.85, CI: –0.94–0.78) (Table 2). 

**Table 2:** Characteristics of the responders and the nonresponders.

	Responder(n = 30)	Non-responder (n = 40)	P
Age, year (median, min–max)	41 (23–79)	40 (19–80)	0.48
Sex Female, n (%) Male, n (%)	18 (60%) 12 (40%)	26 (65%) 14 (35%)	0.66
Plus IVIG, n (%)	13 (43%)	23 (57%)	0.24
PLT in the diagnosis, × 109/L (mean, SD)	17.6 (11.4)	24.7 (19.04)	0.07
MPV in the diagnosis, fL (mean, SD)	11.09 (3.12)	9.38 (1.76)	0.005
PLT after the treatment, × 109/L (mean, SD)	149.8 (115)	22.9 (18.4)	< 0.001
MPV after the treatment, fL (mean, SD)	9,32 (1.82)	9,4 (1.76)	0.85
P*	0,004	0,928	

PLT: platelet, L: litre, MPV: mean platelet volume, fL: femtoliter, IVIG: Intravenous immunoglobulin.

Assessing the whole study population, it was found that the MPV value significantly changed after the treatment (10.11 fL to 9.37 fL, P = 0.014, CI: 0.15–1.33). By analysing the responder group and the nonresponder group separately; there was a significantly decrease of the MPV in the responder group after the treatment, while there was not any significant change in the nonresponder group (P = 0.004 CI: 0.62–2.91; P = 0.92 CI: –0.52–0.47, respectively) (Table 2).

The results of those analyses show that MPV value was higher in the diagnosis in the responder group and also it decreased after the treatment in the responders while it did not change in the nonresponder group (Table 2).

### 3.4. Predictive role of the MPV in the diagnosis 

As the MPV was higher in the responder group in the diagnosis and it decreased after the treatment, we questioned whether the MPV in the diagnosis may be used for the prediction of the treatment response or not.

**Figure 2:**
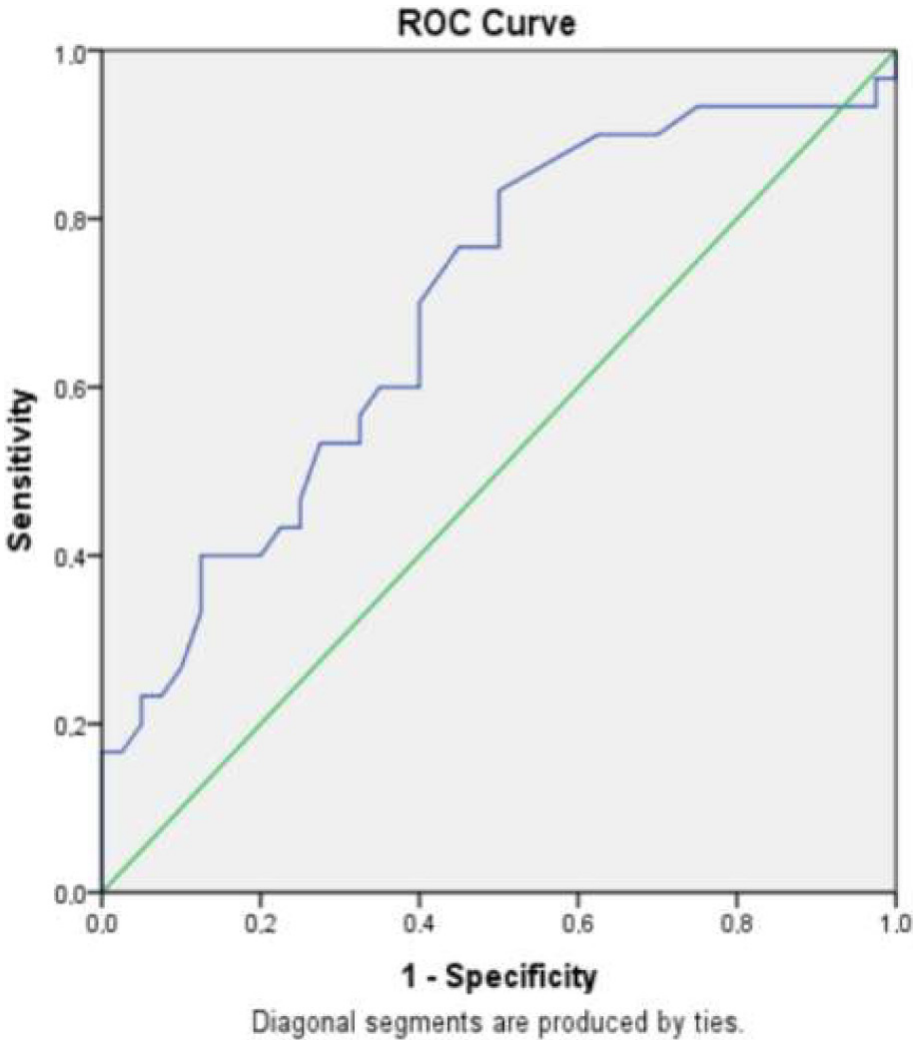
ROC curve of the MPV in the diagnosis and the response AUC: 0.69.

The ROC curve analysis was implemented for the MPV in the diagnosis and the response to the first line therapy. The area under the curve (AUC) was 0.69 (P = 0.006, CI: 0.56–0.82) (Figure 2). The sensitivity for the responders was calculated for MPV > 8.85 fL (sensitivity: 83.3%, specificity: 50%, negative predictive value (NPV): 80%). The specificity was calculated for MPV > 11.15 fL (sensitivity: 40%, specificity: 80%, positive predictive value (PPV): 60%). For the cut-off value of 12.5 fL for the MPV, the specificity was 97.5% and PPV was 83.3%. For the cut-off value of 13 fL, the specificity and the PPV was 100%. In this cut-off value of > 13 fL, odds ratio was 8.99 for the responders (P = 0.003).

## 4. Discussion

In this study, the objective was to evaluate the relationship between the MPV and response to the first line treatment in newly diagnosed adult ITP patients. Response rate was similar to the previous studies [14,15]. MPV was higher in the diagnosis in the responders and also it decreased after the treatment in the responders while it did not change in the nonresponders. When the MPV is higher than 13 fL, the specificity and positive predictive value were 100% for the response, odds ratio was 8.9.

A few previous studies have investigated the relationship between the MPV and ITP. MPV was found higher in ITP than aplastic anaemia [3], chemotherapy-related myelosuppression [4], myelodysplastic syndrome [8] and other hypoproductive thrombocytopenia [6]. MPV was also found higher in ITP than healthy controls [6,12]. However, diagnostic criteria of the ITP, confounding factors and excluding criteria have not been indicated in these studies. One study with 81 patients shows that MPV is normalized after first line treatment and is gradually increasing before the first and second relapses and is normalized again after the second line treatment [9]. But the patient population of this study is heterogeneous and response data of the patients are not certain. Another study with the 233 newly diagnosed ITP patients shows a nonlinear relationship between MPV and ITP relapse. The MPV was an independent risk factor of the ITP relapse when it is less than 21 fL [10]. Both of these studies take into account the long term follow up, like for 6 months. In daily outpatient practice, it is important to know which patient will respond after 1 to 3 weeks, especially if the patient has bleeding or very low platelet count which can cause life threatening bleeding. In our study, we aimed to predict which patients will respond to the therapy in the next outpatient control which is about 1 to 3 weeks later. We may suggest that if the cut-off is 12.5 fL for the MPV, the higher MPV than this value may predict the response and patient can be followed safely outpatient with high specificity.

The pathophysiology of ITP includes antiplatelet antibodies and dysregulated immune system, especially T cells. The 60% to 70% of the patients have antibodies which cause platelet destruction and impaired megakaryopoiesis. Patients having no antibodies have abnormal T cells that cause platelet destruction or diminish production [1]. Each pathologic mechanism plays varying roles in each patient and because of this pathophysiologic heterogeneity, it has not been possible so far to predict the mechanism from clinically available information and to integrate this information with treatment decisions [16].

It is thought that peripheral platelet destruction leads premature and large circulation platelets because of the increased production [7]. So, it is assumed that MPV is high in the ITP patients. The previous studies indicated that MPV may be used to differentiate between hypo and hyper productive thrombocytopenia and ITP patients have higher MPV than the hypoproductive thrombocytopenia (aplastic anaemia, myelodysplasia) [3–8]. However, different mechanisms may be dominant in different patients and this may cause different MPV values in patients.

Corticosteroids suppress reticuloendothelial phagocytic function and reduce antibody production, leading to a reduced platelet destruction [17]. The IVIG inhibits platelet phagocytosis, suppresses antiplatelet antibody production and neutralizes autoantibodies [18]. Corticosteroids cause a decrease in IgG and IgA levels in 2–4 weeks of the treatment and no change in IgM levels [19,20]. Thus, main therapeutic mechanism of corticosteroids in the ITP is their inhibitory effect on antibody mediated platelet destruction [17].

By thinking that the first line therapy primarily effects antibody mediated platelet destruction [17] and the MPV can be different according to the underlying mechanism of the ITP; it may be stated that the MPV may predict the underlying dominant mechanism and forms an indirect relationship between the underlying mechanism and the treatment response. According to our results, we hypothesize 2 subgroups of the ITP patients: the first line therapy is beneficial in patients whose dominant mechanism is destruction of the platelets. These patients have higher level of MPV in the diagnosis (because of the destruction of the platelets and newly produced large circulating platelets) and the MPV decreases after the response (when destruction stops). The second group is that the underlying mechanism is the impaired production. These patients have a low level of MPV in the diagnosis and does not response to the first line therapy and the MPV does not change after the treatment.

The strengths of our study were strict including and excluding criteria. Because the time interval among the measurements was short, the heterogeneity and incorrect data were decreased. It was aimed to suggest a solution for a daily practical problem. Moreover, our study can arise a new hypothesis about the subpopulations of the ITP patients according to pathogenesis and the treatment response. 

The limitation of our study is its retrospective design and the low sample size. The study does not show the long-term relapse and the second line treatment data. 

In conclusion, the MPV may be used as a predictor of the early response to the first line treatment in the newly diagnosed adult primary ITP patients. Future studies can validate or reject these results by using a larger sample size and search for the relationship between the underlying mechanism, MPV, and the treatment response. 

## Acknowledgements

We would like to thank all the professors and specialists of the Department of Haematology for their help, guidance, contributions, and patient follow-up procedures.
